# A countrywide analysis of 27 solar power plants installed at different climates

**DOI:** 10.1038/s41598-021-04551-7

**Published:** 2022-01-14

**Authors:** Talat Ozden

**Affiliations:** 1 Department of Electrical and Electronics Engineering, Gümüşhane University, Gümüşhane, Turkey; 2 The Center for Solar Energy Research and Applications, Middle East Technical University, ODTÜ-GÜNAM, Ankara, Turkey

**Keywords:** Photovoltaics, Power stations

## Abstract

The world is still heavily using nonconventional energy sources, which are worryingly based on carbon. The step is now alternative energy sources hoping that they will be more environmentally friendly. One of the important energy conversion forms by using these sources is photovoltaic solar systems. These type of power plants is on the increase in everyday on the world. Before investment a solar power plant in a specified region, a techno-economic analyse is performed for that power plant by using several meteorological data like solar irradiance and ambient temperature. However, this analyses generally lacks evaluation on effects of climatic and geographical conditions. In this work, 5 years of data of 27 grid-connected photovoltaic power plants are investigated, which are installed on seven different climate types in Turkey. Firstly, the power plants are categorized considering the tilt angles and Köppen–Gieger climate classification. The performance evaluations of the plants are mainly conducted using monthly average efficiencies and specific yields. The monthly average efficiencies, which were classified using the tilts and climate types were from 12 to 17%, from 12 to 16% and from 13 to 15% for tilts 30°/10°, 25° and 20°, respectively. The variation in the specific yields decrease with elevation as y(x) =  − 0.068x + 1707.29 (kWh/kWp). As the performances of photovoltaic systems for some locations within the Csb climatic regions may relatively lower than some other regions with same climate type. Thus, techno-economic performance for PVPP located in this climate classification should be carefully treated.

## Introduction

Photovoltaic (PV) energy conversion is the leading renewable energy resource toward a more sustainable future. Its global potential is much higher than that of other renewables^1–3^. In addition, it is now very cost-effective due to its economic dawn scale^4,5^. Today’s total global investment is larger than 600 GW. China is in the first place, having more than 200 GW installments^6^. Rooftop solar plants are also increasing considerably, together with building-integrated PV (BIPV) and many other niche products^7^. Interestingly, research and developments on solar energy and solar PV to increase efficiencies and decrease costs continue to achieve more environmentally friendly useful energy^8,9^. Therefore, there are many research topics of interest to reach better efficiencies and better installation schemes. Thus, a summary of some recent works on crystalline utility-scale PV power plants (PVPPs) is as follows.

In a recent review article by Kabir et al.^3^, the potential and prospects of solar energy, the benefits, and limitations are discussed in detail. Outlining the cost-effectiveness due to dawn economic scales, they concluded that solar energy technology is one of the most promising renewable energy sources to meet the future global energy demand.

Performance and degradation analyses were carried out for large-scale grid-connected solar PVPPs in the semiarid tropical environment of India by Malvoni et al.^10^. They obtained an overall efficiency of 11% for PVPP and a performance ratio of 74.73%. They reached the degradation rate using the least square regression, Holt-Winters seasonal model, and classical seasonal decomposition methods. The authors also compared the degradation of mono-Si PV systems for various locations in the Indian climate.

There are now relatively long-term data on solar PVPPs to be analyzed to obtain information on feasibilities for their yield increase and future investments. Plants are constructed in different climate areas worldwide, and there are some recent works on this topic covering some climates.

Dash et al.^11^ classified the climates of India to reach criteria based on the performance of PVPP. They used the indoor properties of four different modules having different PV cells using a solar simulator. The measurements are carried out at different irradiance values and different module temperatures conducted in an environmental chamber. I–V characteristics are measured to obtain the properties of maximum power, open-circuit voltage, short-circuit current, voltage, and current at maximum power. Using the measurements, they proposed seven zones of India’s climates based on the annual daytime irradiance and ambient temperature. They used the proposed statistical analysis of Gasparin et al.^12^ for the I–V curve parameters of the photovoltaic modules.

Cubukcu and Gumus^13^ analyzed the outcomes of a 2130.7 kWp grid-connected power plant constructed in eastern Anatolia, which has run since 2016. They used 1-year data from January to December 2017. Monthly, seasonal and yearly analyses are carried out, and their simulation results are close to the real-time measured values, slightly small. Significant differences are obtained seasonally, with an expected peak value in summer and the opposite in winter. They also compared their findings with photovoltaic power plants located at different sites worldwide (Mauritania, Crete, Singapore, Algeria, Malawi, Oman, and India). Significant differences are obtained in the performance ratio and the other parameters that they attribute to local environmental characteristics and rated power.

Daher et al.^14^ evaluated 4 years of operation of a power plant in a desert environment using the IEC 61724 standard. They estimated the impact of climate using a combination of analysis techniques. They reached an average performance ratio of 90% for PV arrays and 84% for the global grid-connected system with corresponding monthly average efficiencies of 12.68% and 11.75%. A cone-shaped seasonal variation in PV module efficiencies is obtained with a sharp maximum in July. The authors also evaluated the impact of ambient temperature and soiling-induced losses with a reduction in the performance ratio by 0.7% for each 1 °C rise in daily ambient temperature. The soiling losses vary between 0.03% following rainfall events and 14.23% during dry, dusty periods.

A recent article by Shorabeh et al.^15^ evaluated power plant site selection in different climates for Iran. They used a GIS-based multicriteria decision analysis using the ordered weighted averaging (OWA) technique to generate maps for potential locations utilizing sustainable energy resources for four provinces with different climates of Iran. Their results show that the areas in an arid climate, such as the Yazd region, contain more suitable sites for solar power plants than wet climate provinces. The sensitivity with OWA shows that the criterion “fault” has the minimum effect. In contrast, the criteria “slope” and “road network” have the maximum impact on the area of the highly desirable class.

Dabou et al.^16^, in relatively earlier work for 2010, concentrate on the weather conditions for an on the grid-connected photovoltaic system installed in the Saharan area of south Algeria (Adrar) having high ambient temperature, low humidity rate, and summer strong solar irradiance values. Daily-based analysis and the data carried out in the Unit of Research in Renewable Energy were conducted under various daily climatic conditions (clear, cloudy, and sandstorm days). Their results show that due to system losses, the lowest values are in the performance ratio (76.5% clear day), efficiency (10.88%), and inverter (1.18 h/day) due to the high average ambient temperature of 32.3 °C. They attributed their findings to high module temperature on clear days (41.1 °C) and the fast-changing solar irradiance caused by variations in cloud cover or dust storm effects.

Othman et al.^17^ stated that solar irradiance uses under vastly changing climatic disturbances should be accounted for in construction; mathematical modeling is almost impossible because of the physical phenomena relating to the climate. They add that the current and future sustainable energy production of PV systems will be affected by the supply. They used deep learning to predict the development of the future potential of any geographic site. This algorithm allows improvements in decision-making concerning solar PV or concentrating PV (CPV) plants. Using the data of NASA and the Tunisian National Institute of Meteorology, they achieved the climatic parameters with a case study region of El Akarit, Gabes, Tunisia, for 1985. The calculation results suggested an increase in production from the case study region, as was confirmed by the 2019 measures. They underline deep learning algorithms instead of conventional calculation methods since they state that traditional approaches based on measurements obtained using hardware solutions (ground sensors) are expensive and difficult to implement.

Dahmoun et al.^18^ evaluated the performance of a grid-connected PVPP (installed power: 23.92 MWp) in Algeria. They studied on the measured data covering 36 months from March 2017 to February 2020. The performance ratio of the PVPP was calculated as over 80%, and the degradation rate of the same PVPP was determined as 0.76%/year in the same period. They also presented various performance results come from different climatic zones to compare this PVPP’s performance results.

One of the recent articles by Gopi et al.^19^ presented a new performance modeling to analyze and model the effect of weather impact on performance of a utility-scale PVPP in a tropical region. They verified their model measured performance data from a 2 MW PVPP. In the other recent article (2021)^20^, they also presented a performance analysis of the same PVPP from during the measured period from 2018 to 2019. They determined that the PVPP performance substantially influences in the tropical monsoon climate conditions. This negative effect may reaches up to 35%.

In the present study, the available measured data from 27 solar PVPPs installed almost all over the country land area, which has different climatic zones. To the best of my knowledge, this study is the first extended experimental analysis of many plants to deduce prospects for research and development. The investigation is mainly carried out using two different parameters of the PV systems as the efficiency and specific yield. Comparative analysis is conducted, and the results are discussed and explained in detail. It should be noted that the laboratory results of the properties of the systems are considerably different compared to outdoor measurements^21–27^. This study aims to provide the performance results and evaluation methodology for 27 PVPPs for the first time. The PVPPs are classified and analyzed considering their tilt angels and site climatic conditions. Thus, the study on the specific yield of PVPPs is carried out taking into account the Köppen-Gieger climate classification and elevation. A linear and realistic methodology is provided to foresee the future of solar/renewable energy to enlighten the prospects.

The second part of the article is the presentation of data in hand, and the limitations are given and explained. Climate classifications of the country and the developed methodology to handle the PVPP data, the climate zones, and application details are also shown in this section. The results and discussion are given next, with proposed precautions for the existing plants and recommendations for the planned PVPPs. The last part presents the conclusion and the subsequent research interest on long-term performance analysis using the data in hand.

## Materials and methods

### Regulations of PVPP in Turkey

PVPP regulations of the country can be divided into two legislative streams: the first stream is named the supporting mechanism for renewable energy investments (YEKDEM), and the other stream is countryside renewable energy investments (YEKA).

The YEKDEM has two different types of applications: licensed and unlicensed investments. To obtain licensed applications, the company should win a tender under negotiations during the process. The investment can be larger than 1 MWe, which is more profitable than smaller constructions. The pricing per kWh of electricity distribution companies under regulations can continue for 15 years. There are 36 PVPP devices, approximately 400 MWe installed all along with the country. There is no tender for the other application type (unlicensed), and the procedure applies to the distribution company to be chosen according to satisfying the application regulations. This type of application must be ≤ 1 MWe and endure on-gridding for at most 10 years. Both of the applications are based on feed-in-tariff schemes. A total of 7000 PVPPs of approximately 6000 MWe are installed along with the country within the second type of YEKDEM installation by the end of January 2021^28^.

The second (YEKA) is mainly based on the tendering procedure of decreasing the price per kWh of the applying companies upon competing. The investment of PVPP installation area is given by governmentally displaced lands of different regions of the country. On-gridding of the investment taken under tendering conditions can be at most 15 years. This tendering procedure will be conducted within the coming period.

There is also a new regulatory scheme for rooftop and BIPV installations. Satisfying the conditions, electricity producers can have the right to construct the installment. The pricing per kWh of an on-grid installment is based on netting producers stated by the distribution company over 10 years.

### Data and analyses

In the present study, the data obtained and analyzed are from 27 installations YEKDEM PVPP of ≤ 1 MWe. Since the data taken are collected from different companies using different instrumentational techniques, detailed analyses and treatment should be conducted as described in the following.

There is no measuring device for incoming solar irradiation, other climatological parameters, or module temperature on some PVPP investments. Although some of the devices are mounted on some of the plants, they do not give accurate measurement results. Some plants do not have precise measuring instrumentations, such as old Si diode sensors^21^. Such data are not utilized in the present work.

All PVPP data are obtained from three source supplying firms. It should be noted that the measured energy production data are obtained from readings of the electronic electricity meters. The data of 22 plants are taken from the operating and maintenance organization company Sunsis^29^. The other data are obtained from Saha Energy^30^ and Meysa Energy^31^ firms, which are 2 and 3 of the plants, respectively. Solar irradiation data on tilted PV modules obtained from the firms have missing monthly values, and in some of the months, there are missing daily values to reach monthly values. Information on these data is given in Table A2 of the supplementary dataset. In addition, for 16 of the plants, one year or more of tilted solar irradiation data were not measured. Therefore, to obtain the tilted values of solar irradiation, Eq. (1) Using the horizontal global solar irradiation data taken by the Turkish State Meteorological Service (TSMS) stations, close stations that have proper measured data were located. The following are considered when choosing these stations:

- The distance of the meteorological station (MS) is less than 100 km.
- The altitude of MS is similar to PP installed area.
- PP and MS have the same climatological classification.

In these analyses, it is noticed that the tilted data calculated using TSMS measurements compared to the existing measurements on plants highly deviate in the months with higher cloudiness. For example, Table 1 gives the data of the Nevsehir-3 plant for the months November to February for 2019.

**Table 1:** Deviation and percentage error of tilted plants measured and calculated from TSMS data for Nevsehir-3.

Months (2019)	Tilted irradiation (measured) (kWh/m^2^)	Tilted irradiation (calculated) (kWh/m^2^)	Error (%)
January	94.62	68.96	37
February	103.76	89.81	16
March	154.84	144.48	7
April	131.59	130.47	1
May	177.89^a^	187.08	− 5
June	188.16	189.97	− 1
July	212.57	216.60	− 2
August	199.57	199.50	0
September	171.40	167.62	2
October	142.80	136.16	5
November	107.27^b^	94.17	14
December	64.27	41.68	54
Total/average (error)	1,748,74	1666.51	11

^a^There are missing data for 1 day for this month.

^b^There are missing data of 4 days for this month.

The PVPP regulations for the country allow the investment with DC power rating to be larger than the AC power rating up to 20%. However, the plant cannot supply on-grid electricity more than the AC power rating of PVPP. This situation results in less than possible electricity supply since AC power becomes less than DC power due to inverter clipping.

The yield and measured irradiation data obtained from PVPPs were not considered, if there might be a considerable missing number of days. The months with only one day of missing data were used by inserting the average data value of that specific month. Whenever the calculated solar irradiation data were needed, the data were used only between April and September months, since the calculations significantly deviated for the other months, as presented in Table 1.

### Power plants and meteorological stations

**Figure 1:**
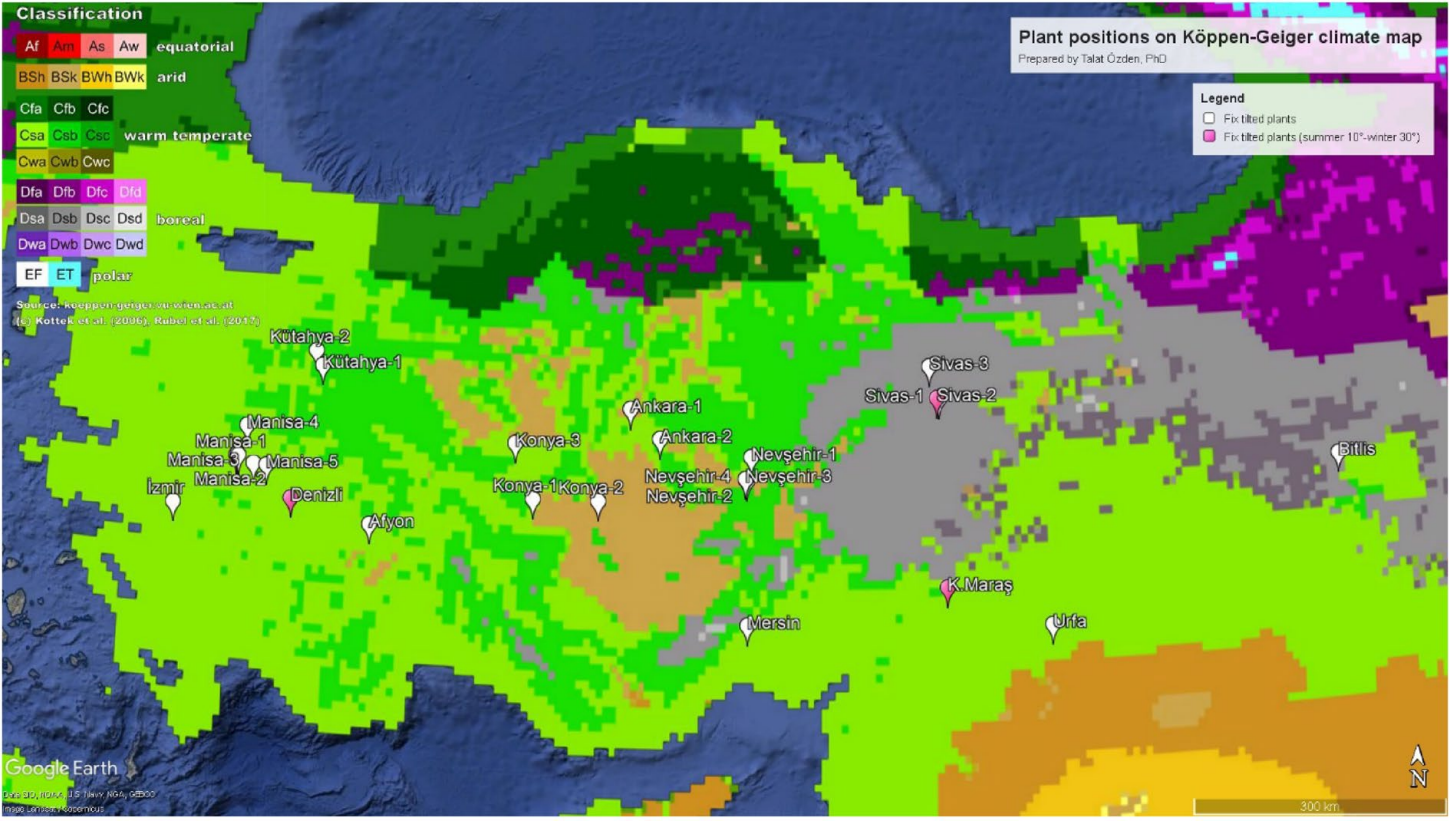
Location of solar power plants on the Köppen-Geiger climate classification map (Google Earth Pro and http://koeppen-geiger.vu-wien.ac.at/present.htm).

PVPPs of Anatolia (Minor Asia) have many PVPPs installed over many differing land areas. The climate types of Turkey are 14 in terms of Köppen–Geiger climate classification, although they are collected within three typologies: arid, temperate, and cold. In the northeastern Black Sea high altitude zone and a short land area of the high southern mountains, the classification is accepted as polar tundra. The map and climate classification of the country are depicted in Fig. 1^32–34^. The descriptions and explanations of the categories are well documented and agreed upon at an international level. The short names of the climate classifications appear on the left high box of the map. The details can be followed from references^34^, and a shortened description is in the following.

The climates classified by Köppen–Geiger and later slightly modified by Beck et al.^33,34^ can be explained shortly as the temperate (C) and cold (D) climates are distinguished using a 0 °C instead of a threshold of 3 °C, which was suggested by Russell^35^. The arid (B) subclimates were mainly identified depending on the relative humidity, and 70% of precipitation occurred in summer or winter. Third, the sub-climates dry summer (S) and dry winter (W) within the C and D climates are contradictory because more precipitation falls in winter than in summer and otherwise assigns W. The details of the climatological concerns can be followed using Beck et al.’s article^34^. It is good to state again that Anatolia has heavily diversified climate types, so a critical region is to be investigated. The categorization of the climate schemes will be itemized in “Results and discussion” section.

Given the above explanations for climate classifications, names and information on the PVPPs are presented in Table 2. Thus, achievable trustworthy investigation results can be deduced. PVPP power plants and the 3-monthly values and yearly averages of the parameters of interest are tabulated. The overall results are in accord with the experimental findings presented in the corresponding literature. It should be noted that the evaluated data cover measures globally, and meaningful direct site analyses can be deduced for a higher renewable energy transition of the Globe.

**Table 2:** Locations, elevations, and distances between them of solar PV plants and stations.

Solar power plants					Meteorological stations					Distance^b^ (km)
Name	Latitude	Longitude	Climate Classification^a^	Elevation (m)	Name	Latitude	Longitude	Climate Classification^a^	Elevation (m)	
Manisa-1	38.581	28.316	Csa	297	MS-1	38.464	27.370	Csa	208	83
Manisa-2	38.591	28.301	Csa	307	MS-1	38.464	27.370	Csa	208	82
Manisa-3	38.515	28.519	Csa	909	MS-1	38.464	27.370	Csa	208	100
Manisa-4	38.880	28.406	Csa	856	MS-1	38.464	27.370	Csa	208	101
Manisa-5	38.509	28.689	Csa	652	MS-1	38.464	27.370	Csa	208	115
Manisa-6^c^	38.582	28.286	Csa	178	MS-1	38.464	27.370	Csa	208	82
İzmir	38.067	27.500	Csa	121	MS-1	38.464	27.370	Csa	208	45
Denizli^c^	38.199	29.025	Csa	844	MS-2	37.434	28.860	Csa	1190	85
Mersin	37.122	34.762	Csa	990	MS-3	37.231	34.737	Bsk	1033	153
K. Maraş^c^	37.473	37.228	Csa	812	MS-4	37.462	37.080	Csa	693	115
Afyon	37.993	30.046	Csa	1010	MS-2	37.434	28.860	Csa	1190	121
Urfa	37.076	38.530	Csa	713	MS-4	37.462	37.080	Csa	693	32
Konya-3	38.856	31.849	Csa	1003	MS-5	38.369	31.430	Csa/Csb	1002	65
Ankara-1	39.215	33.285	Csa	1162	MS-6	39.079	33.066	Csa	1005	24
Ankara-2	38.931	33.660	Csa/Csb/BSk	1100	MS-6	39.079	33.066	Csa	1005	53
Konya-1	38.318	32.089	Csa/Csb/BSk	1049	MS-5	38.369	31.430	Csa/Csb	1002	58
Kütahya-1	39.661	29.249	Csb	793	MS-7	39.817	28.655	Csa/Csb	654	62
Kütahya-2	39.52	29.342	Csb	781	MS-7	39.817	28.655	Csa/Csb	654	53
Nevşehir-2	38.533	34.748	Csb	1390	MS-8	38.616	34.702	Csb	1260	10
Nevşehir-3	38.553	34.731	Csb	1454	MS-8	38.616	34.702	Csb	1260	8
Nevşehir-4^c^	38.553	34.731	Csb	1454	MS-8	38.616	34.702	Csb	1260	8
Nevşehir-1	38.766	34.801	Csb/Csa	1091	MS-8	38.616	34.702	Csb	1260	18
Konya-2	38.315	32.904	BSk	1041	MS-9	37.861	32.584	Csa/Bsk	1011	58
Sivas-1	39.316	37.160	Dsb	1740	MS-10	38.725	36.390	Dsb	1542	93
Sivas-2^c^	39.322	37.134	Dsb	1813	MS-10	38.725	36.390	Dsb	1542	92
Sivas-3	39.632	37.047	Dsb	1651	MS-10	38.725	36.390	Dsb	1542	115
Bitlis	38.588	42.325	Dsb/Csa	2027	MS-11	38.585	42.264	Dsb	2550	5

^a^The classification is determined by using the Köppen–Geiger climate classification map.

^b^This value shows the distance as the crow flies between MS and PVPP.

^c^Tilt angle is set to 10 degree in April and 30 degree in September for these PVPPs.

**Figure 2:**
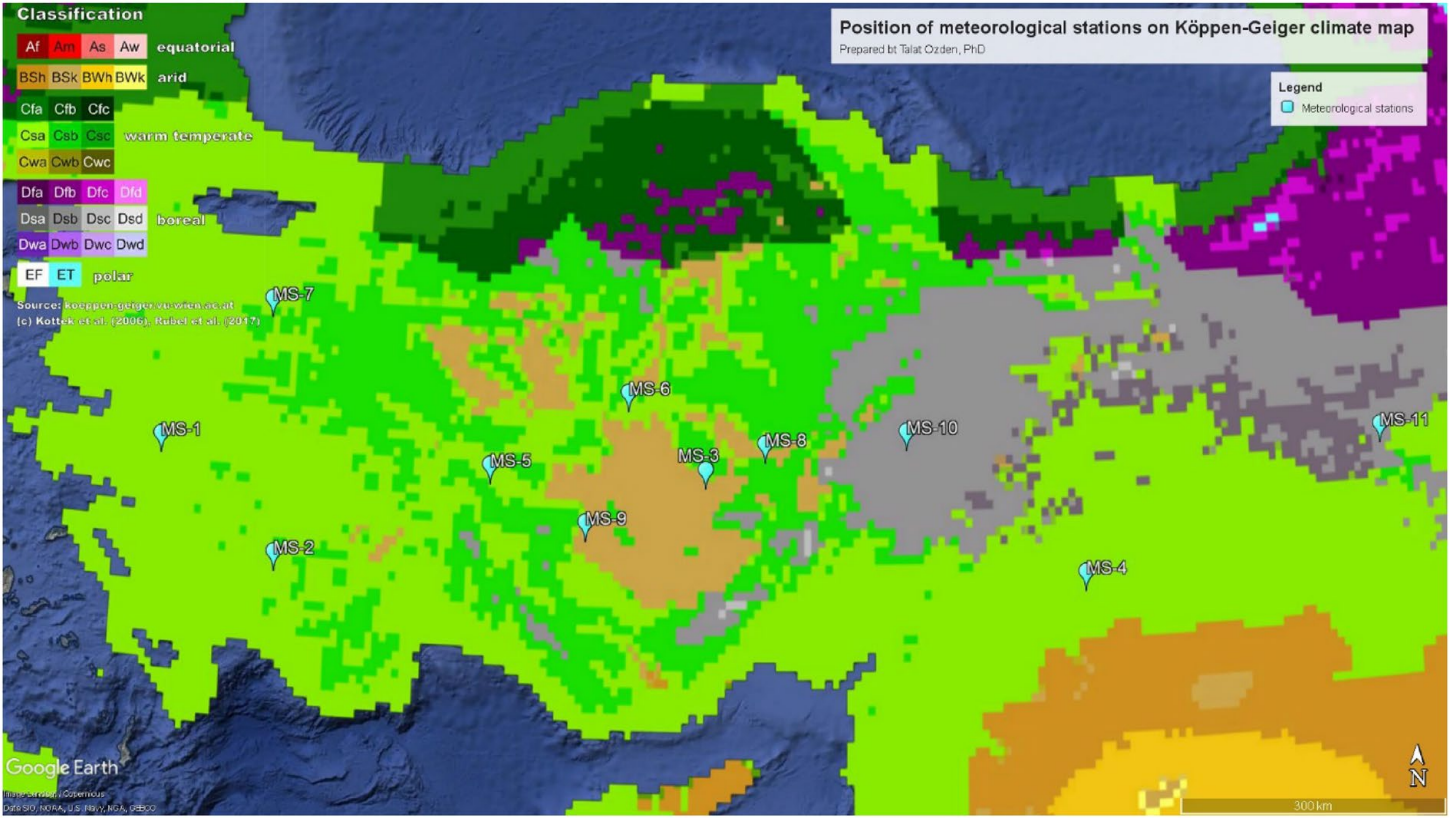
Location of meteorological stations on the Köppen–Geiger climate classification map (Google Earth Pro and http://koeppen-geiger.vu-wien.ac.at/present.htm).

The PVPP data, which are diverse in the climate zones, should be handled using whether the data (mainly obtained from the TSMS) are close to the analyzed systems. Therefore, it should be good to present the data that are received and used in another map, as given in Fig. 2. Information about the locations is tabulated in Table 1 above. The data obtained should carefully be handled to reach scientifically utilizable measurements of investigation. Therefore, they are all evaluated so that well-documented long-term data series are obtained and used.

### Method

The main issue is to make proper estimations of the monthly average solar irradiance to form a base for techno-economic analysis. In some of the articles^18^, the subject is discussed in detail, and within the experimental scientific measures of accuracy, the following isotropic equation for the monthly average of daily solar irradiation, ${\overline{H} }_{t}$^36^, for a tilted surface can be used:

1 $${\overline{H} }_{t}={\overline{H} }_{b}{\overline{R} }_{b}+{\overline{H} }_{d}\left(\frac{1+\cos\beta }{2}\right)+\overline{H}{\rho }_{g}\left(\frac{1-\cos\beta }{2}\right).$$

The monthly average daily solar irradiation $\overline{H }$ on a horizontal surface is measured by the State Meteorological Offices. Direct and diffuse components incident on a horizontal surface ${\overline{H} }_{b}$ and ${\overline{H} }_{d}$ can be estimated^37^. ${\overline{R} }_{b}$ is the ratio due to tilt with respect to horizontal, *β* is the tilt angle and ${\rho }_{g}$ is the ground reflectance for the site. It should be noted that the experimental data used to calculate $\overline{H }$ are checked if trustworthy in terms of their values and comparing them with the theoretical findings.

The mathematical methodology is as the followings. The monthly outdoor efficiency can be expressed as^21^:

2 $$\eta =\frac{{\sum }_{j}^{N}{\left({\overline{E} }_{out}\right)}_{j}}{{\sum }_{j}^{N}{\left({\overline{H} }_{t}\right)}_{j}}.$$

The total monthly yield of PVPP is ${\overline{E} }_{out}$, and the total tilted irradiation on the PV modules is ${\overline{H} }_{t}$. The efficiency is based on hourly measured values averaged over a month, and note that the denominator is obtained using hourly measured horizontal surface data as explained above. The resulting equations are obtained to lie within the ranges of experimental observations.

Specific yield (${Y}_{s}$) can be obtained as of yearly total using:

3 $${Y}_{s}=\frac{{\sum }_{j}^{N}{\left({\overline{E} }_{out}\right)}_{j}}{{P}_{STC\_DC}}.$$

## Results and discussion

The climate types of the regions in which PVPPs are installed are Csa, Csb, BSk, and Dsb. In summary, to clarify, they can be described as follows^33,38^:

- BSk: Arid, Steppe, and Cold.
- Csa: Warm temperate, Dry summer, and Hot summer.
- Dsb: Snow climate, Dry summer, and Warm summer.
- Csb: Warm temperate, Dry summer, and Warm summer.

**Figure 3:**
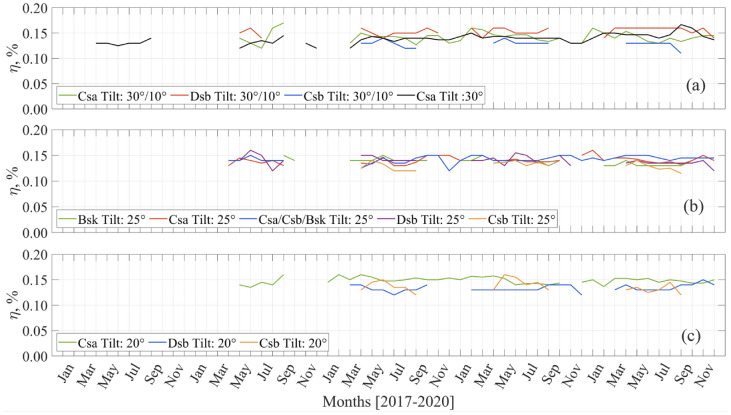
Monthly average efficiencies, which are classified using the tilts and climate types (**a**) Tilt: 30°/10° and Tilt: 30°, (**b**) Tilt: 25°, (**c**) Tilt: 20°.

Figure 3 gives monthly efficiency values for the power plants categorized using their tilt angles and local climates. The efficiency values are the averages of 3 years of monthly results for the same climates and having the same tilt power plants. The data of some months are missing due to either missing relevant records or the derived solar irradiation of ${I}_{t}$ from horizontal irradiation taken from distance TSMS stations.

As shown in Fig. 3a, the power plants in the Csb climate have weaker monthly efficiency values than the others. In the Dsb climate, monthly efficiencies seem better, attributed to dry summers and colder regions of the country (see Fig. 1). The situation is then a valid argument to be used in installations of PVPP. Figure 3b is only for PVPP of 25°. There are 5 monthly averages of 12 climatically classified PVPP efficiencies. As can be observed, PVPP located at Csa and Csa/Csb/BSk climates seems to be better than the others. Secondly, Dsb is the next. Bsk is the third in the range, but these PVPP monthly efficiencies seem close to Csb-located PVPPs. In Fig. 3c, there are only 20° tilt PVPP in the three different climate types. Similar to Fig. 3a,b, PVPPs located in Csa are better, while Csb and Dsb average monthly efficiencies are weaker.

Table 3 gives the specific yields of PVPPs under consideration and the climate classification of the region of installation. It should be noted that these values are unified to provide the specification of PVPPs constructed on the corresponding climatic sites. A simple analysis can be carried out for Manisa-1 and Manisa-2, in which plants have similar climate and construction properties (Table 1, Table A1 in Supplementary Dataset). Manisa-2 has specific yields less than Manisa-1 for the years 2017–2020, which should be due to the lower module and inverter efficiencies of Manisa-2. In addition, the number of inverters for Manisa-2 is higher than that for Manisa-1, which results in more power losses mainly due to cabling and connectors.

**Table 3:** Specific yields of the PVPPs.

PP name	Climate classification^b^	Specific yields (kWh/kWp)				
		2016	2017	2018	2019	2020
Manisa-1	Csa	1123.97^c^	1632.02	1564.57	1621.98	1677.17
Manisa-2	Csa	1650.30	1607.19	1550.21	1603.52	1643.94
Manisa-3	Csa	0.00	1085.59^c^	1586.29	1643.89	1688.34
Manisa-4	Csa	0.00	1072.78^c^	1601.16	1643.66	1674.92
Manisa-5	Csa	0.00	116.53^c^	1467.32	1586.86	1611.91
Manisa-6^a^	Csa	0.00	1350.68^c^	1591.95	1648.30	1687.27
İzmir	Csa	0.00	0.00	1627.72	1642.58	1675.29
Denizli^a^	Csa	0.00	188.61^c^	1644.54	1640.32	1700.82
Mersin	Csa	0.00	1468.29^c^	1550.22	1503.49	1644.28
K. Maraş^a^	Csa	0.00	14.46^c^	1546.45	1607.15	1648.38
Afyon	Csa	687.00^c^	1675.30	1620.24	1653.36	1709.19^d^
Urfa	Csa	0.00	0.00	0.00	1300.53^c^	1635.64
Konya-3	Csa	0.00	1507.34^c^	1573.69	1558.33	1622.32
Ankara-1	Csa	0.00	148.08^c^	1506.93	1552.12	1657.92
Ankara-2	Csa/Csb/BSk	0.00	149.99^c^	1591.29	1596.04	1661.05
Konya-1	Csa/Csb/BSk	0.00	1487.55^c^	1583.70	1558.77	1633.97
Kütahya-1	Csb	0.00	0.00	1103.65^c^	1419.94	1296.09
Kütahya-2	Csb	0.00	0.00	1295.23^c^	1411.42	1390.35
Nevşehir-2	Csb	0.00	27.96^c^	1586.71	1618.80	1641.48
Nevşehir-3	Csb	0.00	0.00	1533.01^c^	1551.46	1576.63
Nevşehir-4^a^	Csb	0.00	0.00	1578.63	1633.54	1660.70
Nevşehir-1	Csb/Csa	0.00	9.53^c^	1561.52	1578.74	1612.85
Konya-2	BSk	0.00	635.41^c^	1655.62	1663.27	1713.97
Sivas-1	Dsb	0.00	1255.85^c^	1595.86	1672.80	1606.66
Sivas-2^a^	Dsb	0.00	1156.07^c^	1608.95	1655.84	1718.51
Sivas-3	Dsb	0.00	0.00	1514.20	1545.33	1547.95
Bitlis	Dsb/Csa	0.00	0.00	0.00	1459.33	1507.14

^a^Tilt angle is set to 10 degree in April and 30 degree in September for these PVPPs.

^b^The classification was determined by using a Köppen–Geiger climate classification map.

^c^There are missing months/days in the indexed years since the operation of plants does not start from the beginning of the year.

^d^DC installed power was increased in this PVPP after October 2020 [for details, see Table A1 in Supplementary Dataset]. Therefore, the specific yield in 2020 is higher than that in 2019.

Manisa-1 and -2 have a tilt angle of 30° while Manisa-3 and -4 have a tilt angle of 20° for the modules. The 20° plants had considerably larger specific yield values than the 30° tilted module plants. All these plants are in the same climate, while for Anatolia, the optimal tilt angles should be between 28° and 32°, but the 20° PVPP of Manisa-3 and Manisa-4 have higher specific yields than Manisa-1 and Manisa-2. Thus, this situation can be attributed to approximately one-point higher module efficiencies of Manisa-3 and Manisa-4 (η ~ 16.5%, Table A1 in Supplementary Dataset). This determination yields higher energy generation for these plants if a higher tilt value of approximately 28°–32° would be used. However, due to the restriction of the land area, 20° tilt that the companies are using. That is, they might be placing the arrays with shortened distances in between.

The Nevsehir-4 plant arrays have two different tilt angles of 10° and 30° in 1 year for seasonal performance increases. At the same time, the Nevsehir-3 plant is on the same land and has a fixed tilt of 20°. All the other properties of the plants were the same (Table A1 in Supplementary Dataset) and comparable. Specific yields for these plants (Table 3) show that Nevsehir-4 is always larger by approximately 80 kWh/kWp (this difference corresponds to an energy yield difference of approximately 80 MWh for one year). It should be noted that the variation of the tilt angle from 10° to 30° is not suitable for optimal generation. The land area optimal fixed tilt angle is approximately 30° for 1 year; therefore, the seasonal variation in the tilt angle should be between 20° and 40°.

Specific yield values obtained using reliable yearly data give consistent results. Therefore, the average values of the PVPP-specific yield of each climate and tilt (Table 4) can be analyzed to reach scientific arguments. The highest results from 2020 are 1718.51 kWh/kWp at the Dsb climate type and with twofold adjustable tilt positions. However, the tilt of PVPP is constant at 25°, and the result is 1713.97 kWh/kWp in the BSk climate type, which is very close to the findings for Dsb. Therefore, it could be stated that a twofold adjustment for PVPP in BSk would be much better than the specific yield obtained in Dsb.

**Table 4:** Average specific yields of PVPP considering the Köppen–Gieger climate classification and tilts.

No	Climate type	Tilt (°)	# of PVPPs	Specific yield (kWh/kWp)		Δ Specific yield (%)
				2019	2020	
1	BSk	25	1	1663.27	1713.97	3.0
2	Csa	30/10	3	1631.92	1678.82	2.8
3	Csa	30	3	1594.61	1647.81	3.2
4	Csa	25	3–4^a^	1595.65	1661.76	4.0
5	Csa	20	4	1629.25	1662.62	2.0
6	Csa/Csb/BSk	25	2	1577.41	1647.51	4.3
7	Dsb	30/10	1	1655.84	1718.51	3.6
8	Dsb	25	2	1566.07	1556.90	− 0.6
9	Dsb	20	1	1545.33	1547.95	0.2
10	Csb	30/10	1	1633.54	1660.70	1.6
11	Csb	25	3	1539.16	1516.81	− 1.5
12	Csb	20	2	1481.44	1483.49	0.1

^a^There is no yearly all data for Urfa PVPP in 2019. Therefore, this PVPP was extracted from the average specific yield calculation for 2019.

It should be noted that the specific yield decreases or increases over the years depending on the solar irradiation values and is indirectly dependent on the degradation rate due to the types of equipment.

Additionally, note that for the two-fold adjustment tilt PVPP of Csa, Dsb and Csb have better specific yields. The difference for the Dsb and Csb climate types is approximately 150 kWh/kWp, which is relatively high, but for Csa, the difference is 20 kWh/kWp, which is significantly smaller.

The PVPPs in the climates of Csb, Csa/Csb/BSk, and Csa ranked from smaller specific yields to higher values. The reason is that the climate of the central part of Anatolia (Fig. 1) has higher precipitation and generally lower altitudes below 1000 m. These regions, in general, are forestry and greener. In other words, Csb has a warm summer compared to Csa, so the precipitation and relative humidity can be higher some regions of Csb. The lower specific yield values for Csb can be attributed to lower solar irradiation due to these higher climatic conditions. For example, average yearly horizontal total solar irradiation from 2017 to 2020 for Kütahya-1 and -2 PVPP regions, which are located in Csb climate type, is 1395 kWh/m^2^. Additionally, for the same regions, average yearly precipitation is 524 mm/year in the same period. However, the others PVPPs regions in the climates of Csb (Nevşehir-2, -3 and -4) have higher solar irradiation values (1600 kWh/m^2^) and have lower precipitation (144 mm/year) more than Kütahya site. Therefore, investors should be careful while choosing a PVPP area in the Csb climatic regions.

The specific yields for 2020 are, in general, better than those for 2019. The situation can be attributed to their climatological states, which are different.

A final note is on Nevsehir-3 and -4. These plants are at the same location (see Table 2), module type, inverter, and DC installed power, but the tilt angles are different, 20° and 30°/10° for Nevsehir-3 and -4, respectively. These differences result in approximately 84 kWh/kWp in terms of specific yields for Csb climate classification.

**Figure 4:**
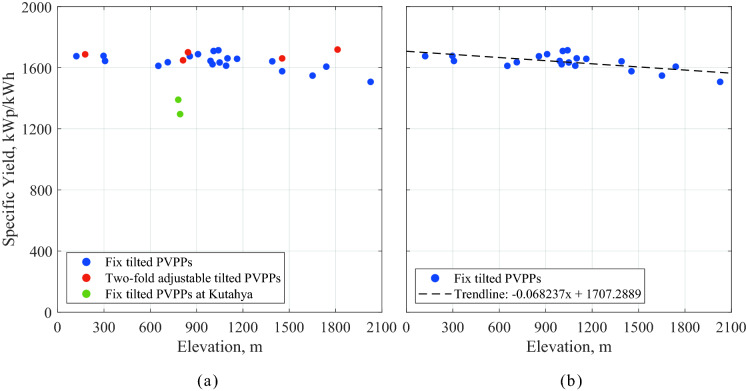
Variation in specific yield with elevation (**a**) for all PVPPs, (**b**) Only the fixed tilted PVPPs except the two PVPPs at Kutahya.

Figure 4 shows the specific yield of PVPPs with respect to elevation. As can be observed, PVPPs with a twofold adjustable tilt (30°/10°) instead have higher specific yield values (the red dots, Fig. 4a). The two green dots have weaker specific yields than all others, attributed to the higher number of missing days due to grid connection problems.

Given these findings, it would be non-truthfully to discuss the variation of specific yields with elevation using the data of all PVPPs. Therefore, the PVPPs with fixed tilt angle data are presented with a trendline in Fig. 4b. A decrease in the specific yield with elevation can be observed, and the Pearson correlation coefficient is determined to be − 0.6385. This is moderate negative correlation. In addition, it can be stated that the scattering of the data points is mainly due to small tilt angle differences and climatic issues.

## Conclusions

Outdoor measurement data of approximately 5 years of 27 utility-scale grid PVPPs in Anatolia are examined. They are located in seven different climates of the land and have diverse characteristics. The performance of PVPPs is determined, and technical discussions are given and outlined. Regions with even different climate types are investigated. Monthly average efficiencies and specific yields are mainly analyzed. The monthly average efficiencies, which were classified using the tilts and climate types, were from 12 to 17%, from 12 to 16% and from 13 to 15% for tilts 30°/10°, 25° and 20°, respectively. Specific yields essentially decrease with elevation with a linear regression of y(x) =  − 0.068x + 1707.29 (kWh/kWp). In obtaining this expression, five twofold and two fixed-tilt PVPPs are excluded. The final note is that the techno-economic results for such power plants should be carefully treated as the performance of PVPPs in the some Csb regions could be lower than PVPPs installed some other regions of Csb.

## Supplementary Information

Supplementary Tables.
